# Effect of Functional Group-Modified UiO-66 on the Dehydrogenation of Ammonia Borane

**DOI:** 10.3390/molecules30071487

**Published:** 2025-03-27

**Authors:** Senliang Xi, Dawei Xu, Renzeng Chen, Wenhao Yao, Wenying Wu, Teng Zhang, Liang Yu

**Affiliations:** 1 Key Laboratory of Cluster Science Ministry of Education, Beijing Key Laboratory of Photoelectronic/Electrophotonic Conversion Materials, Advanced Research Institute of Multidisciplinary Science, School of Chemistry and Chemical Engineering, Beijing Institute of Technology, Beijing 100081, China; xisenliang1996@163.com (S.X.); xudaweispark@163.com (D.X.); 3220215241@bit.edu.cn (R.C.); ywh200012@163.com (W.Y.); wwy15933833065@163.com (W.W.); 2 Advanced Technology Research Institute (Jinan), Beijing Institute of Technology, Jinan 250000, China

**Keywords:** ammonia borane, metal–organic frameworks, nanoconfinement, dehydrogenation, functional group

## Abstract

Ammonia borane (AB) has attracted much attention in the field of solid-state hydrogen storage due to its high hydrogen storage capacity. Nanoconfinement in UiO-66 can reduce the hydrogen release temperature. In particular, terephthalic acid was used as a linker to further improve the dehydrogenation properties through the modification of -NH_2_, -OH, -NO_2_, -Br, and -F groups. The hydrogen release content of 0.5AB/UiO-66 was 1.98 wt.%, whereas the hydrogen release content of UiO-66-2OH modified by -OH groups increased to 3.85 wt.%. The non-covalent interaction results show that -NH_2_ and -OH preferred adsorption with -BH_3_, and the H in -NH_2_ and -OH were able to interact directly with the H in AB to modify the dehydrogenation process of AB, whereas -NO_2_, -Br, and -F indirectly affected the charge density of hydrogen atoms in AB to alter the dehydrogenation property of AB. The modification of functional groups provides a theoretical basis for the design of high-performance MOF nanoconfinement AB composite hydrogen storage materials.

## 1. Introduction

Hydrogen is a very promising renewable energy source due to its high energy density and environmental friendliness [1]. However, the primary obstacle to the more widespread utilization of hydrogen is the question of its storage and delivery [2,3]. There is a pressing need to develop safe and efficient means of hydrogen storage that are superior to the current methods of high-pressure gaseous and low-temperature liquid storage [4,5]. One potential solution is to develop efficient materials for hydrogen storage. These include, for example, liquid organic hydrogen carriers [6,7], metal hydrides [8,9,10], alkali-metal coordinated hydrides [11,12,13], and boron and nitrogen hydrides [14,15,16,17,18]. In recent years, AB has received considerable attention as a promising solid hydrogen storage material, exhibiting an ultra-high theoretical gravimetric hydrogen storage density (19.6 wt.%) [19,20], along with a lower dehydrogenation temperature compared to other materials, such as NaBH_4_ [21,22] and MgH_2_ [9,23].

Nevertheless, pure ammonia borane (AB) is not the optimal choice for chemical hydrogen storage, because under heating conditions, complex BNH_x_ components are formed and impurity gases are produced, including ammonia, diborane, and borazine [24,25]. Furthermore, AB exhibits a reduced capacity to dehydrogenate a single molecule of hydrogen at low temperature (200 °C), which presents a challenge to the effective use of AB within the hydrogen economy.

Due to their advantageous properties, including a uniform pore structure, structural diversity, adjustable pore size, and a high specific surface area [26,27,28], metal–organic framework (MOF) materials have demonstrated significant efficacy in the domain of nanoconfinement. Numerous studies have indicated that the nanoconfinement capabilities of MOF materials can substantially enhance the dehydrogenation performance of AB, such as AB@MOF with JUC-32-Y as a porous framework, as reported for the first time by Li et al. [29]. The analytical results showed that some of the Y^3+^ are unsaturated sites and JUC-32-Y has the uncoordinated functional group C=O. The presence of Y^3+^ and the functional group has the potential to alter the charge distribution in the AB, resulting in the chemical bonding in the AB becoming unstable [30,31,32,33]. This destabilization would reduce the peak dehydrogenation temperature of AB@JUC-32-Y to 84 °C without the generation of volatile impurities. However, the effect of unsaturated metal sites and functional groups on AB dehydrogenation was not analyzed by Li et al. Gao et al. [34] investigated the effect of different functional groups on the dehydrogenation properties of AB. The ligand was modified on the basis of MIL-101(Cr) by adding -NH_2_, -NO_2_, and -NHCOCH_3_ functional groups. The results indicated that the incorporation of various functional groups significantly lowered the dehydrogenation temperature and enhanced the dehydrogenation capacity of AB, with the -NH_2_ group exhibiting the most obvious effect. First-principles calculations demonstrated that the charge densities of the hydrogen atoms in AB were altered following the introduction of the -NH_2_ group into the ligand. The incorporation of functional groups resulted in a modification of the charge density of AB, subsequently enhancing its dehydrogenation performance. Peil et al. [35] further investigated the effect of functional groups on the dehydrogenation of AB with the help of solid-state NMR. Two functional groups, -NH_2_ and -OH, were added to Al-MIL-53, and a significant decrease in the dehydrogenation temperature of AB was observed with the addition of these functional groups. The decomposition pathways of the two materials are very similar, with Al-MIL-OH producing 31% cubic boron nitride and 69% boron oxide at 140 °C, while 23% cubic boron nitride and 77% boron oxide are detected for Al-MIL-NH_2_. It is speculated that the high percentage of boron oxide may be due to the presence of -OH. However, the interaction between AB and different functional groups remains unclear.

Consequently, this study investigates the impact of various functional groups, -NH_2_, -OH, -NO_2_, -Br, and -F, on the properties of AB when incorporated into UiO-66. The purity of hydrogen and the dehydrogenation content of AB/UiO-66-X (X = NH_2_, OH, 2OH, NO_2_, Br, F) were examined using temperature-programmed desorption mass spectrometry (TPD-MS) and temperature-programmed desorption gas chromatography (TPD-GC). Additionally, the interactions between AB and the functional groups were analyzed utilizing XPS and the first principles investigation. This study provides theoretical guidance for the design of high-performance MOF nanoconfined AB composite hydrogen storage materials.

## 2. Results and Discussion

**Figure 1:**
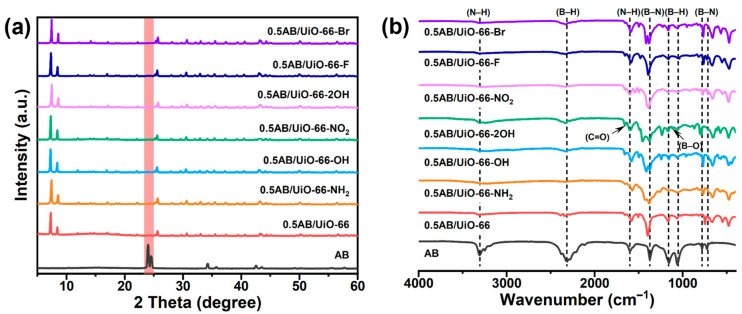
(**a**) Characterization of 0.5AB/UiO-66 and 0.5AB/UiO-66-X. PXRD patterns, (**b**) FT-IR spectra.

The characterization of UiO-66 and UiO-66-X are shown in the Supplementary Materials. Figure 1a and Figure 1b show the PXRD patterns and the FT-IR spectra of 0.5AB/UiO-66 and 0.5AB/UiO-66-X, respectively. In Figure 1a, the PXRD patterns of 0.5AB/UiO-66 and 0.5AB/UiO-66-X do not show any peaks of pristine AB, suggesting that the AB particles are very small and well distributed. In Figure 1b, the peaks observed in the range of 3310–3210 cm^−1^ are associated with the antisymmetric stretching of H-N bonds. The absorption peaks at 2312 cm^−1^ and 2275 cm^−1^ are ascribed to the stretching of H-B bonds. Additionally, the peaks at 1597 cm^−1^, 1553 cm^−1^, and 1375 cm^−1^ correspond to the scissors modes of H-N. The stretching mode of B-N is identified at a peak of 778 cm^−1^, while the H wagging modes are detected at peaks of 1055 cm^−1^ and 728 cm^−1^ [36]. Additionally, a weak absorption peak associated with B-O is noted in the 0.5AB/UiO-66-2OH sample, suggesting the presence of O-H and the binding of AB.

The N_2_ adsorption–desorption isotherms of 0.5AB/UiO-66 and 0.5AB/UiO-66-X are shown in Figure S5. The specific surface area of the samples was found to be nearly negligible, suggesting that during the impregnation process, the AB component was incorporated into the pores of the MOF.

**Figure 2:**
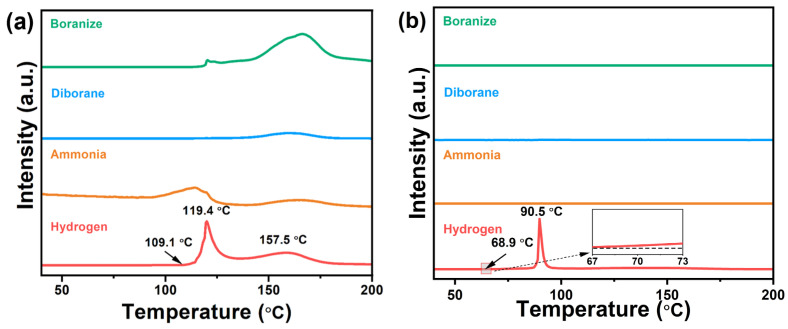
TPD-MS profiles of (**a**) AB and (**b**) 0.5AB/UiO-66.

The dehydrogenation properties of 0.5AB/UiO-66 and UiO-66-X were investigated. As illustrated in Figure 2a, pure AB commenced the release of the first equivalent of hydrogen at approximately 109.1 °C, reaching a peak temperature of around 119.4 °C. The subsequent dehydrogenation event exhibited a peak temperature of approximately 157.5 °C. Concurrently, the dehydrogenation process of pure AB resulted in the generation of significant quantities of volatile gases, including ammonia, diborane, and borazine. Figure 2b shows the TPD-MS profiles of 0.5AB/UiO-66. Their initial and peak dehydrogenation temperatures were lower than those of pure AB. For the initial dehydrogenation temperature, the 0.5AB/UiO-66 is 68.9 °C and the dehydrogenation peak temperature is 90.5 °C. Meanwhile, no production of NH_3_, borazine, or diborane was detected for 0.5AB/UiO-66 during the dehydrogenation process. In addition, due to the nanoconfinement effect of UiO-66, the pore may be restricted to AB during dehydrogenation, resulting in only one step of dehydrogenation of AB.

**Figure 3:**
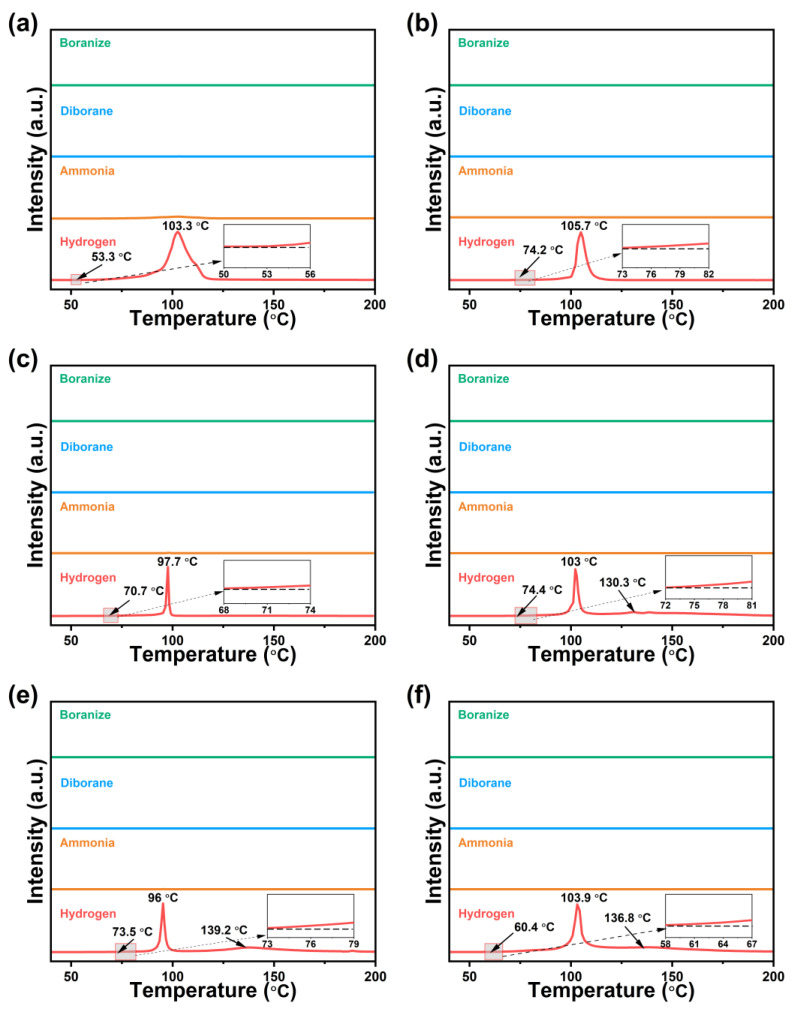
TPD-MS profiles of (**a**) 0.5AB/UiO-66-NH_2_, (**b**) 0.5AB/UiO-66-OH, (**c**) 0.5AB/UiO-66-2OH, (**d**) 0.5AB/UiO-66-NO_2_, (**e**) 0.5AB/UiO-66-F, and (**f**) 0.5AB/UiO-66-Br.

The TPD-MS profiles of 0.5AB/UiO-66-X are shown in Figure 3. The dehydrogenation temperatures of 0.5AB/UiO-66-X were significantly lower compared to those of pure AB. Among the samples, 0.5AB/UiO-66-NH_2_ was observed to produce minimal ammonia during the dehydrogenation process. In contrast, 0.5AB/UiO-66 did not generate any impurity gases during the same process. This observation has led to the hypothesis that the reaction between AB and -NH_2_ in UiO-66-NH_2_ during dehydrogenation with heating may be responsible for the ammonia production. The mechanism of action of the dehydrogenation reaction of -OH and -NH_2_ with AB may be different from that of -NO_2_, -Br, and -F, so that -NO_2_, -Br, and -F cause AB to release the second molecular weight of hydrogen. The mechanism of action for the dehydrogenation of AB by acting on different functional groups is discussed in detail in the latter part of this paper. The higher dehydrogenation temperature of 0.5AB/UiO-66-X compared to 0.5AB/UiO-66 may be due to the stronger interaction of functional groups within the pore directly with AB.

**Figure 4:**
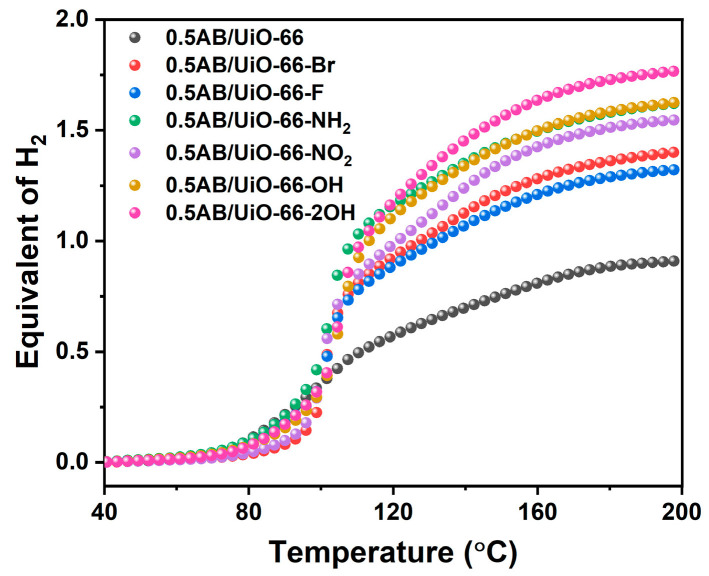
Equivalent of H_2_ evolution during thermolysis of 0.5AB/UiO-66 and 0.5AB/UiO-66-X.

Figure 4 shows the non-isothermal desorption profile of 0.5AB/UiO-66 and 0.5AB/UiO-66-X (X = NH_2_, OH, 2OH, NO_2_, Br, F). It is evident that the AB loaded with MOF exhibits hydrogen release at about 80 °C. The results are presented in Table 1; 0.5AB/UiO-66 can release 0.91 molecular equivalents of hydrogen. After modifying the functional groups, the hydrogen release of 0.5AB/UiO-66-X was higher than that of 0.5AB/UiO-66, and the dehydrogenation of 0.5AB/UiO-66-NH_2_, 0.5AB/UiO-66-OH, and 0.5AB/UiO-66-2OH was significantly higher than that of 0.5AB/UiO-66-NO_2_, 0.5AB/UiO-66-Br, and 0.5AB/UiO-66-F. This may be due to the binding of H in -NH_2_ and -OH with H in AB. Among the composites, the dehydrogenation amount of 0.5AB/UiO-66-2OH reaches 1.77 molecular equivalents, which is significantly higher than that of the other composites. Compared with the results reported in the literature, 0.5AB/UiO-66-2OH released a higher hydrogen content, and although AB-MIL-101-NH_2_ was able to release more hydrogen, its TPD-MS results showed that impurity gases were still released [34].

The XRD patterns of 0.5AB/UiO-66 and 0.5AB/UiO-66-X after complete dehydrogenation are shown in Figure S6a. UiO-66 and UiO-66-X demonstrate the maintenance of a more complete structure. The FT-IR spectra of 0.5AB/UiO-66 and 0.5AB/UiO-66-X after complete dehydrogenation are shown in Figure S6b. The formation of B-O bonds and the disappearance of C=O bonds were observed in all curves, suggesting that AB may react with C=O.

**Figure 5:**
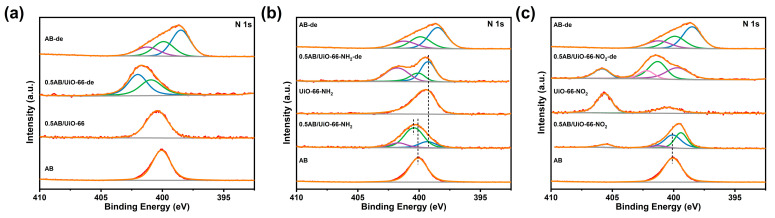
XPS spectra of the N 1s orbitals of the materials. (**a**) 0.5AB/UiO-66 and 0.5AB/UiO-66-de, (**b**) 0.5AB/UiO-66-NH_2_, UiO-66-NH_2_ and 0.5AB/UiO-66-NH_2_-de, (**c**) 0.5AB/UiO-66-NO_2_, UiO-66-OH_2_ and 0.5AB/UiO-66-NO_2_-de.

To investigate the role of different functional groups in the dehydrogenation process, XPS tests on MOFs, 0.5AB/MOFs, and the products after dehydrogenation were performed. As shown in Figure S7, Zr 3d was not significantly shifted before and after MOF loading of AB and after dehydrogenation of AB, indicating that there is no electron transfer between Zr^4+^ and AB. In Figure 5a,b and Figure S8, it can be observed that the N 1s of UiO-66, UiO-66-NH_2_, UiO-66-OH, UiO-66-2OH, UiO-66-Br, and UiO-66-F exhibit a shift towards higher binding energies upon loading AB, indicating an electron-loss state. In contrast, when AB is loaded into UiO-66-NO_2_ (Figure 5c), the binding energy of the N 1s becomes lower, suggesting an electron-gaining state. As illustrated in Figure 5 and Figure S8, the N 1s orbital of 0.5AB/UiO-66 and 0.5AB/UiO-66-X after dehydrogenation exhibited only two peaks near 400.5 eV and 402 eV, corresponding to the B-N bond and N-O bond, respectively. In comparison to the spectra of pure AB, the peak at 398.5 eV was absent, indicating that nano-restricted AB effectively prevents the formation of solid by-products during the dehydrogenation process.

As shown in Figure S9, there is no significant change in B 1s when AB is loaded onto the MOF compared to pure AB. For the 0.5AB/UiO-66 and 0.5AB/UiO-66-X after dehydrogenation, the B 1s peak reveals two peaks, corresponding to the B-N bond and the B-O bond, situated at approximately 192.3 and 193.1 eV, respectively, as previously discussed in the literature [30,38]. Furthermore, the FT-IR spectrum of 0.5AB/UiO-66 and 0.5AB/UiO-66-X after dehydrogenation (Figure S6) also exhibits peaks characteristic of B-N and B-O bonds.

**Figure 6:**
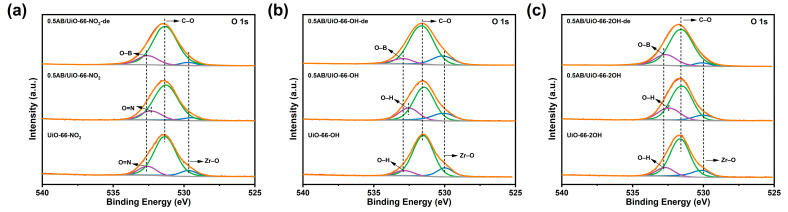
XPS spectra of the O 1s orbitals of the materials. (**a**) 0.5AB/UiO-66-NO_2_, UiO-66-NO_2_ and 0.5AB/UiO-66-NO_2_-de, (**b**) 0.5AB/UiO-66-OH, UiO-66-OH and 0.5AB/UiO-66-OH-de, (**c**) 0.5AB/UiO-66-2OH, UiO-66-2OH and 0.5AB/UiO-66-2OH-de.

As illustrated in Figure 6, the loading of AB into UiO-66-NO_2_, UiO-66-OH, and UiO-66-2OH cause a shift in the O 1s peaks of -NO_2_ and -OH to low binding energies, suggesting an electron-gaining state. Upon full dehydrogenation of AB, the formation of O-B bonds, due to the strong interactions between O and B, improves the dehydrogenation performance of AB. This is mentioned in the published works [35,39].

**Figure 7:**
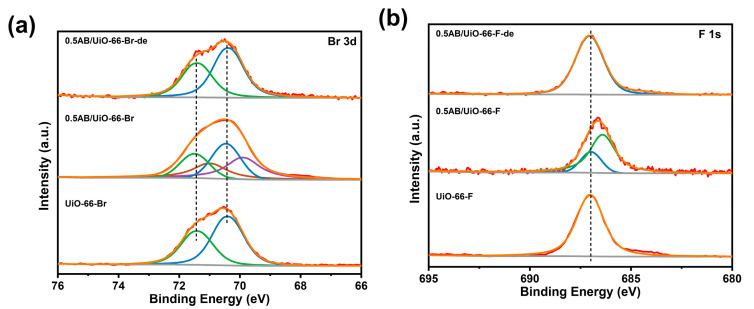
XPS spectra of the (**a**) Br 3d and (**b**) F 1s orbitals of the materials.

Furthermore, as illustrated in Figure 7, the Br 3d orbitals and F 1s orbitals of UiO-66-Br and UiO-66-F exhibited a shift towards lower binding energies following the loading of AB, indicating that an electron transfer occurs between AB and UiO-66-Br and UiO-66-F. However, after the dehydrogenation of AB, the peaks of Br 3d and F 1s orbitals were restored to be consistent with those of UiO-66-Br and UiO-66-F. This suggests that -Br and -F only have an effect on the electronic structure of AB, but are not involved in the reaction during the dehydrogenation of AB.

Non-covalent interaction (NCI) scattering and reduced density gradient (RDG) analyses were conducted to examine and differentiate the interactions [40] between benzene with various functional groups and the compound AB. RDG isosurface mapping contains blue, green, and red regions, standing for the attraction, van der Waals, and steric repulsion, respectively [41].

**Figure 8:**
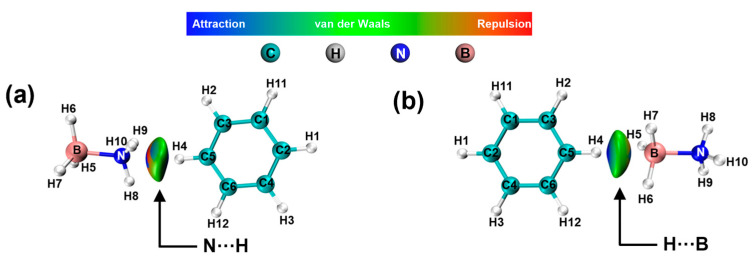
NCI and RDG analysis of AB interactions with -H. (**a**) H···NH_3_-BH_3_, (**b**) H···BH_3_-NH_3_.

The presence of green and a slight amount of blue coloration observed between H_2_BDC and AB, as illustrated in Figure 8a,b, suggests the occurrence of electrostatic interactions between N···H and B···H, respectively. Table S1 illustrates that there is a minimal variation in the charge density of H atoms in AB both before and after the optimization process.

**Figure 9:**
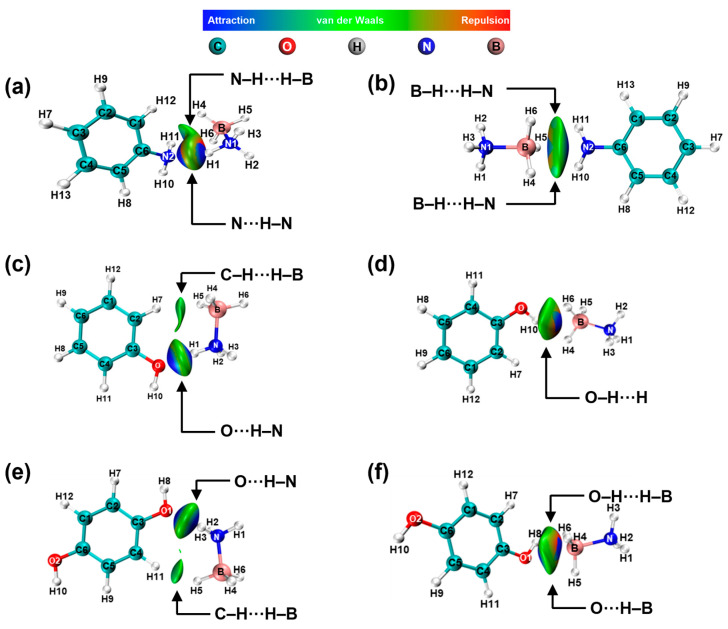
NCI and RDG analysis of AB interactions with -NH_2_ (**a**,**b**) and -OH (**c**–**f**).

The interaction of -NH_2_ with AB is shown in Figure 9a,b. The results indicate the presence of N-H···H-B and N···H-N electrostatic interactions between -NH_2_ and -NH_3_, as well as B-H···H-N electrostatic interactions between -NH_2_ and -BH_3_. When the adsorption site is -NH_3_, the structure is optimized so that -BH_3_ is gradually adsorbed to -NH_2_, whereas when the adsorption site is -BH_3_, the structure is optimized so that -NH_3_ is not adsorbed by -NH_2_, which suggests that -NH_2_ prefers adsorption with -BH_3_ and thus interacts with AB. The same results are also reflected in Figure 9c,d. When the adsorption site is -NH_3_, the structure is optimized so that -BH_3_ is gradually adsorbed to -OH, whereas when the adsorption site is -BH_3_, the structure is optimized so that -NH_3_ is not adsorbed by -OH, which suggests that -OH prefers adsorption with -BH_3_. In addition, the H in both -NH_2_ and -OH can interact electrostatically with the H in AB.

Tables S2–S4 show that in the optimized structure, there is a significant change in the charge density of the H atoms in AB that have electrostatic interactions with -NH_2_ and -OH compared to Table S1.

**Figure 10:**
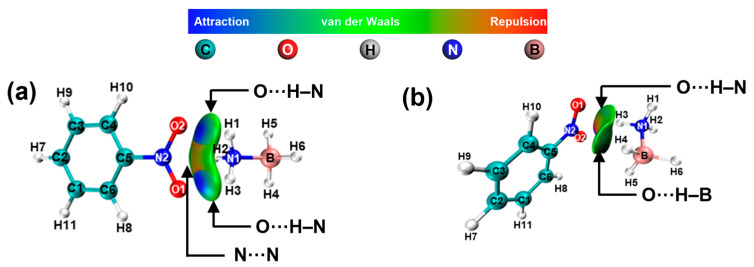
NCI and RDG analysis of AB interactions with -NO_2_. (**a**) NO_2_···NH_3_-BH_3_, (**b**) NO_2_···BH_3_-NH_3_.

In Figure 10, the results indicate the presence of N···N and O···H-N electrostatic interactions between -NO_2_ and -NH_3_, as well as O···H-N and O···H-B electrostatic interactions between -NO_2_ and -BH_3_. In contrast to the -NH_2_ and -OH groups, the -NO_2_ group exerts a lesser influence on the charge density of hydrogen atoms in AB (Table S5).

**Figure 11:**
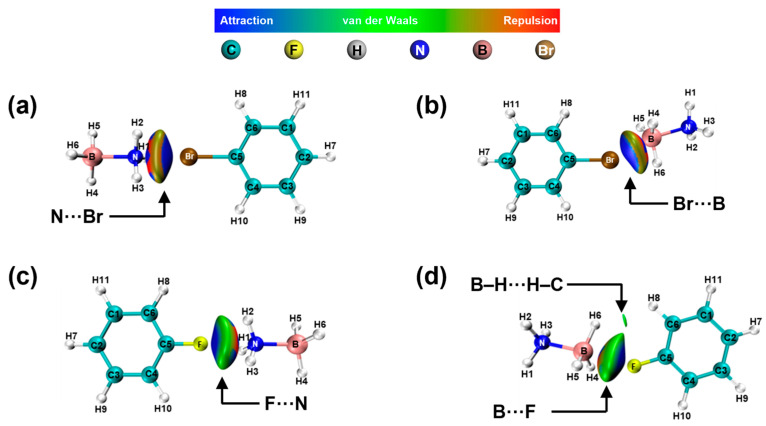
NCI and RDG analysis of AB interactions with -Br (**a**,**b**) and -F (**c**,**d**).

The interactions of the -NH_2_, -OH, and -NO_2_ groups differ from those of the -Br and -F groups. The latter typically engage in electrostatic interactions with N and boron B in AB, which subsequently influences the hydrogen atoms within AB. The results of these interactions are Figure 11a–d. It is observed from Tables S6 and S7 that the change in charge density of H atoms in -NH_3_ is essentially the same, and the change in charge density of H atoms in -BH_3_ is essentially the same.

The NCI results demonstrate that the electronic structure of AB is influenced to varying degrees by the presence of functional groups such as -NH_2_, -OH, -NO_2_, -Br, and -F. All of these functional groups are capable of disrupting the symmetry of the AB structure. It is possible that some of the H atoms in NH_2_-BDC, OH-BDC, and DHTA may interact electrostatically with the H atoms in AB. In light of the observed effect of different functional groups on the extent of AB dehydrogenation (Figure 5), it is postulated that in addition to the release of H atoms from AB, H atoms from some of the linkers may also be released. -NH_2_ and-OH can change the dehydrogenation process of AB in the pore, thus changing the multi-step dehydrogenation process of AB to one-step dehydrogenation. After the dehydrogenation of AB in UiO-66-NO_2_, the peak of the nitro group can still be found in FT-IR (Figure S6b), and it is presumed that the nitro group has not reacted with AB. Similar to -NO_2_, the orbitals of Br 3d and F 1s are consistent with UiO-66-Br and UiO-66-F (Figure 7), respectively, and it is presumed that Br and F also did not react with AB. But -NO_2_, -Br, and -F can activate the second step of the dehydrogenation reaction of AB within the UiO-66-X orifice, as can be illustrated in Figure 3d–f. In conclusion, the electronic structure of AB is affected by the addition of functional groups, which changes the dehydrogenation properties of AB.

## 3. Materials and Methods

### 3.1. Chemicals

Zirconium tetrachloride (ZrCl_4_, 98%) and acetic acid (99.8%) were purchased from Shanghai Aladdin Biochemical Technology Co., Ltd. (Shanghai, China). Ethanol absolute (EtOH, ≥99.5%), N,N-dimethylformamide (DMF, ≥99.5%), and tetrahydrofuran (THF, ≥99.5%) were purchased from Beijing Tong Guang Fine Chemicals Company (Beijing, China). The 1,4-dicarboxybenzene (H_2_BDC, 98%), 2-aminoterephthalic acid (NH_2_-BDC, 98%), 2-hydroxyterephthalic acid (OH-BDC, 98%), 2-bromoterephthalic acid (Br-BDC, 98%), 2-fluoroterephthalic acid (F-BDC, 98%), 2-nitroterephthalic acid (NO_2_-BDC, 98%) and 2,5-dihydroxyterephthalic acid (DHTA, 98%) were purchased from Energy Chemical Co., Ltd. (Shanghai, China). Ammonia borane (NH_3_BH_3_, AB, 97%) was purchased from 9 Ding Chemistry (Shanghai, China).

### 3.2. Instruments and Methods

PXRD patterns were measured using a Rigaku MiniFlex 600 diffractometer (Rigaku Corporation, Tokyo, Japan) with Cu-Kα X-ray radiation (λ = 0.154056 nm). The PXRD patterns were recorded from 3° to 60° (2*θ*) with a step size of 0.02° and a scan rate of 10° min^−1^. FT-IR spectra were collected at a transmission range of 400–4000 cm^−1^ on a Bruker ALPHA spectrometer (Bruker Optik GmbH, Ettlingen, Germany). Nitrogen sorption isotherms were recorded to specific surface areas and pore size distributions by using a Quantachrome Instrument ASiQMVH002-5 analyzer (Quantachrome Instruments, Boynton Beach, FL, USA) at 77 K. The samples were pretreated under a vacuum for 12 h at 120 °C. The pore size distributions were determined by non-local density functional theory (NLDFT) mode. FE-SEM images were collected on a Zeiss SUPRATM 55 SAPPHIRE scanning electron microscope (Carl Zeiss AG, Oberkochen, Germany) with 15 kV voltage. TGA curves were recorded from 40 to 800 °C at a heating rate of 10 °C min^−1^ using a NETZSCH STA 449F5 (NETZSCH, Selb, Germany) under a N_2_ atmosphere (50 mL min^−1^). The samples were pretreated under a vacuum for 12 h at 120 °C. X-ray photoelectron spectroscopy (XPS) measurements were performed on a Thermo Scientific K-Alpha electron spectrometer (Thermo Fisher Scientific, Waltham, MA, USA) using monochromatic Al-Ka radiation of 1486.6 eV. Adventitious carbon was used to calibrate the binding energy shifts of the samples (C 1s = 284.8 eV).

The samples were initially placed in the center of an argon gas flow system (Sevenstar, Beijing, China) (50 mL min^−1^) tube furnace with a ramping rate of 3 °C min^−1^ from room temperature to 200 °C. The decomposition products were analyzed by a Hiden HPR-20 R&D (Hiden Analytical, Warrington, UK). The content of evolved hydrogen was analyzed by an Agilent 7890B using Agilent J&W HP-PLOT Q (Agilent Technologies Inc., Santa Clara, CA, USA). The hydrogen was detected by thermal conductivity detector (TCD) (Agilent Technologies Inc., Santa Clara, CA, USA). GC sampling was performed every 1 min during heating to capture real-time H_2_ concentration. The H_2_ signal was calibrated using standard H_2_/Ar mixtures (1–10 vol%).

The calculation was performed using the Gaussian 09 software (Gaussian Inc., Wallingford, CT, USA) package, using b3lyp/6-31G* functional basis sets [42]. The binding energies of seven molecules, namely terephthalic acid, 2-aminoterephthalic acid, 2-hydroxyterephthalic acid, 2-bromoterephthalic acid, 2-fluoroterephthalic acid, 2-nitroterephthalic acid, and 2,5-dihydroxyterephthalic acid, with the B and N sites of ammonia borane, were calculated separately, and the Mulliken charges of these 14 different combinations were analyzed. Finally, Multiwfn software version 3.7 (Beijing Kein Research Center for Natural Sciences, Beijing, China), combined with the VMD program, was used to perform IGMH analysis on the interactions of these 14 structures and achieve visualization [43,44].

### 3.3. Materials Prepared

Synthesis of UiO-66:

UiO-66 particles were prepared by the reported solvothermal method [45] with modifications. Typically, 63 mg (0.38 mmol) of H_2_BDC and 106 mg (0.45 mmol) of ZrCl_4_ were dissolved in 50 mL of DMF, followed by the addition of 5 mL of acetic acid and sonication. The above solutions were transferred to a Teflon-lined steel autoclave (Yzreactor, Shanghai, China) (100 mL) and heated at 120 °C for 12 h, then cooled naturally to room temperature. The solution was washed three times with DMF to remove unreacted precursors and then solvent exchanged with EtOH over 3 days (1 time per day). Finally, the resulting UiO-66 particles were dried overnight at room temperature in a vacuum oven and then activated under a high dynamic vacuum at 150 °C for 12 h.

Synthesis of UiO-66-NH_2_:

UiO-66-NH_2_ particles were prepared by the reported solvothermal method [46] with modifications. Typically, 81.6 mg (0.45 mmol) of NH_2_-BDC and 105 mg (0.45 mmol) of ZrCl_4_ were dissolved in 45 mL of DMF, followed by the addition of 4.5 mL of acetic acid and sonication. The above solutions were transferred to a Teflon-lined steel autoclave (100 mL) and heated at 120 °C for 12 h, then cooled naturally to room temperature. The solution was washed three times with DMF to remove unreacted precursors and then solvent exchanged with EtOH over 3 days (1 time per day). Finally, the resulting UiO-66-NH_2_ particles were dried overnight at room temperature in a vacuum oven and then activated under a high dynamic vacuum at 150 °C for 12 h.

Synthesis of UiO-66-NO_2_:

UiO-66-NO_2_ particles were prepared by the reported solvothermal method [46] with modifications. Typically, 105 mg (0.5 mmol) of NO_2_-BDC and 117 mg (0.5 mmol) of ZrCl_4_ were dissolved in 50 mL of DMF, followed by the addition of 3 mL of acetic acid and sonication. The above solutions were transferred to a Teflon-lined steel autoclave (100 mL) and heated at 120 °C for 12 h, then cooled naturally to room temperature. The solution was washed three times with DMF to remove unreacted precursors and then solvent exchanged with EtOH over 3 days (1 time per day). Finally, the resulting UiO-66-NO_2_ particles were dried overnight at room temperature in a vacuum oven and then activated under a high dynamic vacuum at 150 °C for 12 h.

Synthesis of UiO-66-Br:

UiO-66-Br particles were prepared by the reported solvothermal method [46] with modifications. Typically, 123 mg (0.5 mmol) of Br-BDC and 117 mg (0.5 mmol) of ZrCl_4_ were dissolved in 50 mL of DMF, followed by the addition of 1 mL of acetic acid and sonication. The above solutions were transferred to a Teflon-lined steel autoclave (100 mL) and heated at 120 °C for 12 h, then cooled naturally to room temperature. The solution was washed three times with DMF to remove unreacted precursors and then solvent exchanged with EtOH over 3 days (1 time per day). Finally, the resulting UiO-66-Br particles were dried overnight at room temperature in a vacuum oven and then activated under a high dynamic vacuum at 150 °C for 12 h.

Synthesis of UiO-66-F:

UiO-66-F particles were prepared by the reported solvothermal method [46] with modifications. Typically, 92 mg (0.5 mmol) of F-BDC and 117 mg (0.5 mmol) of ZrCl_4_ were dissolved in 50 mL of DMF, followed by the addition of 5 mL of acetic acid and sonication. The above solutions were transferred to a Teflon-lined steel autoclave (100 mL) and heated at 120 °C for 12 h, then cooled naturally to room temperature. The solution was washed three times with DMF to remove unreacted precursors and then solvent exchanged with EtOH over 3 days (1 time per day). Finally, the resulting UiO-66-F particles were dried overnight at room temperature in a vacuum oven and then activated under a high dynamic vacuum at 150 °C for 12 h.

Synthesis of UiO-66-OH:

UiO-66-OH particles were prepared by the reported solvothermal method [47] with modifications. Typically, 781 mg (4.3 mmol) of OH-BDC and 1 g (4.3 mmol) of ZrCl_4_ were dissolved in 70 mL of DMF, followed by the addition of 61 mL of acetic acid and 5 mL of H_2_O. The solution was transferred to a three-necked flask and then condensed and refluxed at 120 °C for 15 min, then cooled naturally to room temperature. The solution was washed three times with DMF to remove unreacted precursors and then solvent exchanged with EtOH over 3 days (1 time per day). Finally, the resulting UiO-66-OH particles were dried overnight at room temperature in a vacuum oven and then activated under a high dynamic vacuum at 150 °C for 12 h.

Synthesis of UiO-66-2OH:

UiO-66-2OH particles were prepared by the reported solvothermal method [47] with modifications. Typically, 850 mg (4.3 mmol) of DHTA and 1 g (4.3 mmol) of ZrCl_4_ were dissolved in 70 mL of DMF, followed by the addition of 61 mL of acetic acid and 5 mL of H_2_O. The solution was transferred to a three-necked flask and then condensed and refluxed at 120 °C for 15 min, then cooled naturally to room temperature. The solution was washed three times with DMF to remove unreacted precursors and then solvent exchanged with EtOH over 3 days (1 time per day). Finally, the resulting UiO-66-2OH particles were dried overnight at room temperature in a vacuum oven and then activated under a high dynamic vacuum at 150 °C for 12 h.

Synthesis of AB/MOF:

Here, 100 mg of AB was dissolved in 1 mL THF. Then, 200 mg of MOF was dispersed in 1 mL of THF by ultrasonication for 20 min. The prepared THF solution of AB was added to the above MOF dispersion. Next, the dispersion was continuously stirred for 12 h at room temperature. The sample was put in a vacuum dryer oven at 40 °C overnight for ensuring complete drying. In fact, the mass ratio of AB:MOF was 0.5:1, giving the sample a designation of 0.5AB/MOF, and it was stored in a desiccator for further characterization.

## 4. Conclusions

In this study, the effect of modifying various functional groups (-NH_2_, -OH, -NO_2_, -Br, and -F) in UiO-66 on the thermal dehydrogenation process of AB was investigated. All the functional groups had an effect on the dehydrogenation properties of AB, and the TPD-GC analysis showed that the dehydrogenation ratio of AB was significantly increased by the addition of functional groups compared to UiO-66. Among them, the introduction of -NH_2_ and -OH was able to bring the dehydrogenation of two AB to 3.53 wt.%, which was higher than that of UiO-66 at 1.98 wt.%. The modification of two -OH on terephthalic acid was able to further enhance the dehydrogenation ratio of AB, and UiO-66-2OH increased the dehydrogenation of AB to 3.85 wt.%. The XPS results showed that electron transfer occurred between the modified functional groups and a part of AB. The interactions between these functional groups and AB were investigated by NCI analysis. Overall, for functional groups containing H atoms, they can directly participate in the pyrolytic dehydrogenation reaction of AB by directly interacting with the H in AB, while changing the electronic structure of AB. For functional groups that do not contain H atoms, the dehydrogenation properties of AB can be improved by changing the electronic structure of AB. In this study, the functional group modification of UiO-66 and the effect of different functional groups on AB were analyzed to support the future design of high-performance MOF nanoconfined AB composite hydrogen storage materials.

## Figures and Tables

**Table 1:** Content and equivalent of H_2_ evolution during thermolysis of 0.5AB/MOFs.

Samples	H_2_ Content (wt.%)	Equivalent of H_2_
0.5AB/UiO-66	1.98	0.91
0.5AB/UiO-66-NH_2_	3.53	1.62
0.5AB/UiO-66-OH	3.53	1.62
0.5AB/UiO-66-2OH	3.85	1.77
0.5AB/UiO-66-NO_2_	3.38	1.55
0.5AB/UiO-66-F	2.87	1.32
0.5AB/UiO-66-Br	3.05	1.4
10 wt.%AB/Fe-MIL-53 [35]	1.48	2.26
24 wt.%AB/Al-MIL-53 [35]	1.61	1.03
AB@Cu-BDC [30]	0.88	2.82
AB@Tm(BTC) [37]	0.97	1.94
AB/MOF-5 [31]	1.28	1.41
AB-MIL-101-NH_2_ [31]	5.55	1.7

## Data Availability

The data presented in this study are available upon request from the corresponding authors.

## Supplementary Materials

The following supporting information can be downloaded at: https://www.mdpi.com/article/10.3390/molecules30071487/s1, Figure S1: Characterization of UiO-66 and UiO-66-X. PXRD patterns (a), FT-IR spectra (b), N_2_ adsorption-desorption isotherms (at 77 K) (c) and pore size distributions (d); Figure S2: The SEM image of UiO-66; Figure S3: The SEM images of UiO-66-NH_2_ (a), UiO-66-OH (b), UiO-66-2OH (c), UiO-66-NO_2_ (d), UiO-66-Br (e), and UiO-66-F (f); Figure S4: TGA plots for UiO-66 and UiO-66-X; Figure S5: N_2_ adsorption-desorption isotherms (at 77 K) of 0.5AB/UiO-66 and 0.5AB/UiO-66-X; Figure S6: Characterization of 0.5AB/UiO-66-de and 0.5AB/UiO-66-X-de. PXRD patterns (a), FT-IR spectra (b); Figure S7: XPS spectra of the Zr 3d orbitals of the materials; Figure S8: XPS spectra of the N 1s orbitals of the materials; Figure S9: XPS spectra of the B 1s orbitals of the materials; Table S1: Muliken population analysis of charge densities of AB, H_2_BDC, H_2_BDC-AB; Table S2: Muliken population analysis of charge densities of AB, NH_2_-BDC, NH_2_-BDC-AB; Table S3: Muliken population analysis of charge densities of AB, OH-BDC, OH-BDC-AB; Table S4: Muliken population analysis of charge densities of AB, DHTA, DHTA-AB; Table S5: Muliken population analysis of charge densities of AB, NO_2_-BDC, NO_2_-BDC-AB; Table S6: Muliken population analysis of charge densities of AB, Br-BDC, Br-BDC-AB; Table S7: Muliken population analysis of charge densities of AB, F-BDC, F-BDC-AB; Table S8: The X,Y, and Z coordinates used for computational analysis.
