# Supplementary material for: Effect of Functional Group-Modified UiO-66 on the Dehydrogenation of Ammonia Borane
Source: Molecules. 2025 Mar 27;30(7):1487. doi: 10.3390/molecules30071487 (PMC11990169; doi:10.3390/molecules30071487)
Supplement: Supplementary file 1 [file molecules-30-01487-s001.zip › molecules-3523092-supplementary.pdf]

Effect of functional group-modified UiO-66 on the dehydrogenation of ammonia borane

Supplementary Materials

Figure S1. Characterization of UiO-66 and UiO-66-X. PXRD patterns (a), FT-IR spectra (b), N_2 adsorption-desorption isotherms (at 77K) (c) and pore size distributions (d). P3

Figure S2. The SEM image of UiO-66. P3

Figure S3. The SEM images of UiO-66-NH_2 (a), UiO-66-OH (b), UiO-66-2OH (c), UiO-66-NO_2 (d), UiO-66-Br (e), and UiO-66-F (f). P3

Figure S4. TGA plots for UiO-66 and UiO-66-X (X = NH_2, OH, 2OH, NO_2, Br, F). P4

Figure S5. N_2 ad/desorption isotherms (at 77 K) of 0.5AB/UiO-66 and 0.5AB/UiO-66-X. P4

Figure S6. Characterization of 0.5AB/UiO-66-de and 0.5AB/UiO-66-X-de. PXRD patterns (a), FT-IR spectra (b). P5

Figure S7. XPS spectra of the Zr 3d orbitals of the materials. P5

Figure S8. XPS spectra of the N 1s orbitals of the materials. P6

Figure S9. XPS spectra of the B 1s orbitals of the materials. P7

Table S1. Muliken population analysis of charge densities of AB, H_2BDC, H_2BDC-AB. P8

Table S2. Muliken population analysis of charge densities of AB, NH_2-BDC, NH_2-BDC-AB. P9

Table S3. Muliken population analysis of charge densities of AB, OH-BDC, OH-BDC-AB. P10

Table S4. Muliken population analysis of charge densities of AB, DHTA, DHTA-AB. P11

Table S5. Muliken population analysis of charge densities of AB, NO_2-BDC, NO_2-BDC-AB. P12

Table S6. Muliken population analysis of charge densities of AB, Br-BDC, Br-BDC-AB. P13

Table S7. Muliken population analysis of charge densities of AB, F-BDC, F-BDC-AB. P14

Table S8. The xyz coordinates used for computational analysis. P15

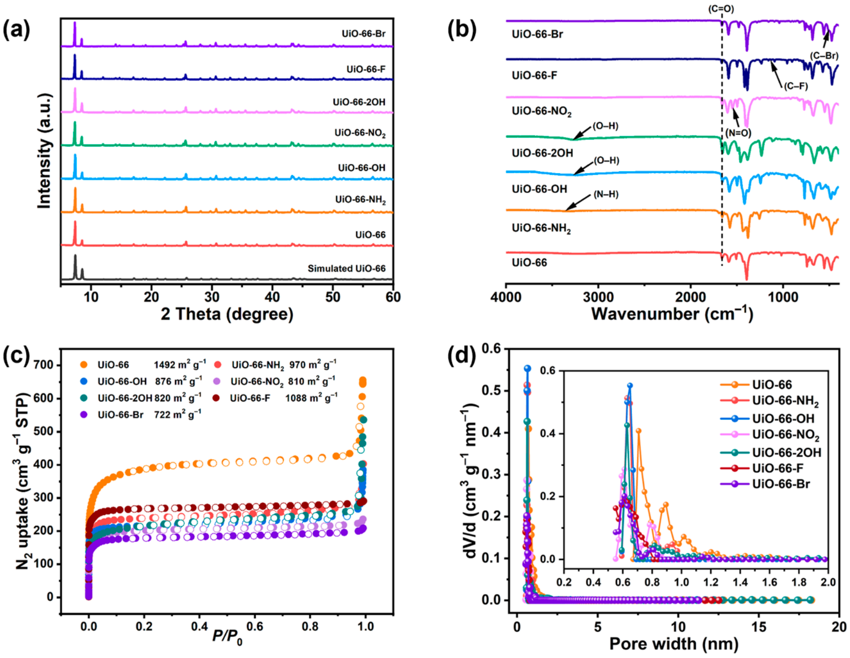

Figure S1. Characterization of UiO-66 and UiO-66-X. PXRD patterns (**a**), FT-IR spectra (**b**), N_2 adsorption-desorption isotherms (at 77K) (**c**) and pore size distributions (**d**).

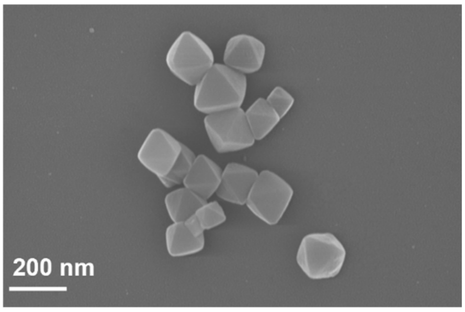

Figure S2. The SEM image of UiO-66.

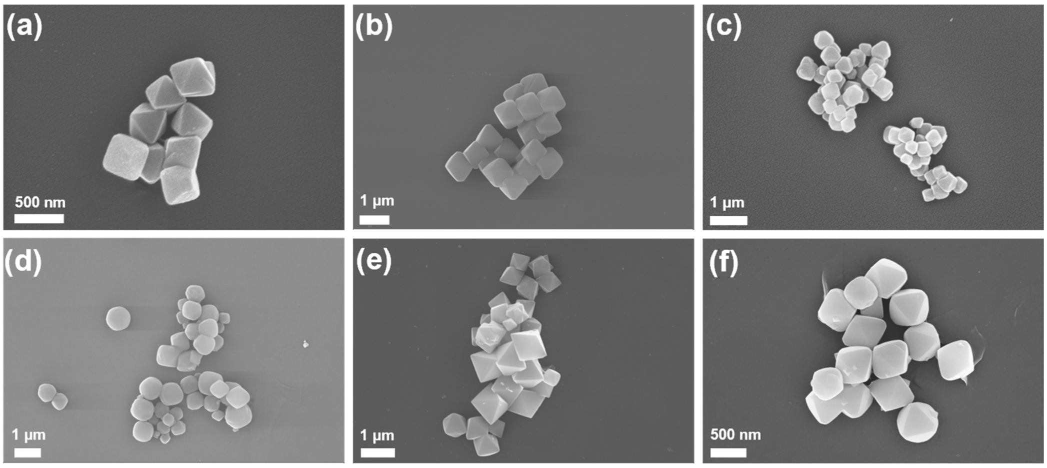

Figure S3. The SEM images of UiO-66-NH_2 (**a**), UiO-66-OH (**b**), UiO-66-2OH (**c**), UiO-66-NO_2 (**d**), UiO-66-Br (**e**), and UiO-66-F (**f**).

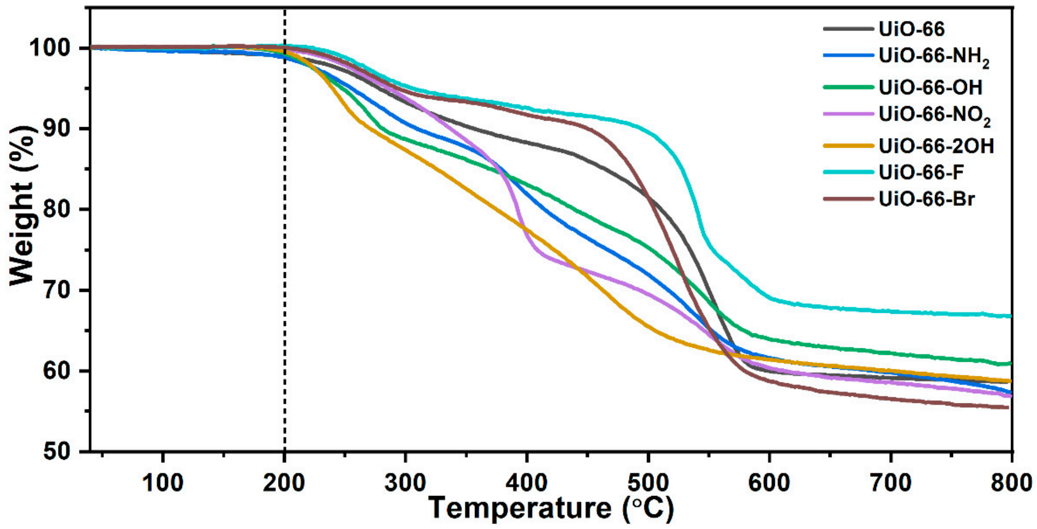

Figure S4. TGA plots for UiO-66 and UiO-66-X.

Figure S1(a) shows the PXRD patterns of UiO-66 and UiO-66-X, and Figure S1(b) shows the FT-IR spectra of UiO-66 and UiO-66-X. In Figure S1(a), UiO-66 and UiO-66-X are in good agreement with the simulated PXRD patterns. In Figure S1(b), the stretching vibrational peak of C=O at 1660 cm^{-1} indicates that UiO-66 and UiO-66-X are unsaturated. The peaks at 3379 cm^{-1}, 3291 cm^{-1}, and 1550 cm^{-1} are the stretching vibration peaks of N–H bond, O–H bond and N=O bond, respectively. The peaks at 1093 cm^{-1} and 511 cm^{-1} are the bending vibrational peaks of C–F and C–Br bonds, respectively. The N_2 adsorption-desorption isotherms and the specific surface area of UiO-66 and UiO-66-X are shown in Figure S1(c). Due to the introduction of functional groups, the specific surface area of UiO-66-X decreases compared to UiO-66. And from the pore size distribution (Figure S1(d)), the pore size of UiO-66-X is obviously smaller than that of UiO-66 after the addition of functional groups. Figure S2 and Figure S3 shows the SEM image of UiO-66 and UiO-66-X, which have typical octahedral structures. The TG curves of UiO-66 and UiO-66-X are shown in Figure S4. All of the materials began to lose weight after 200 °C, indicating that the crystal structures of the materials remained stable up to 200 °C.

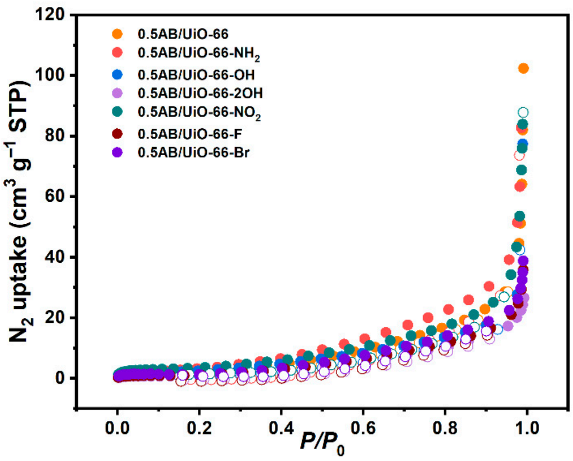

Figure S5. N_2 ad/desorption isotherms (at 77 K) of 0.5AB/UiO-66 and 0.5AB/UiO-66-X.

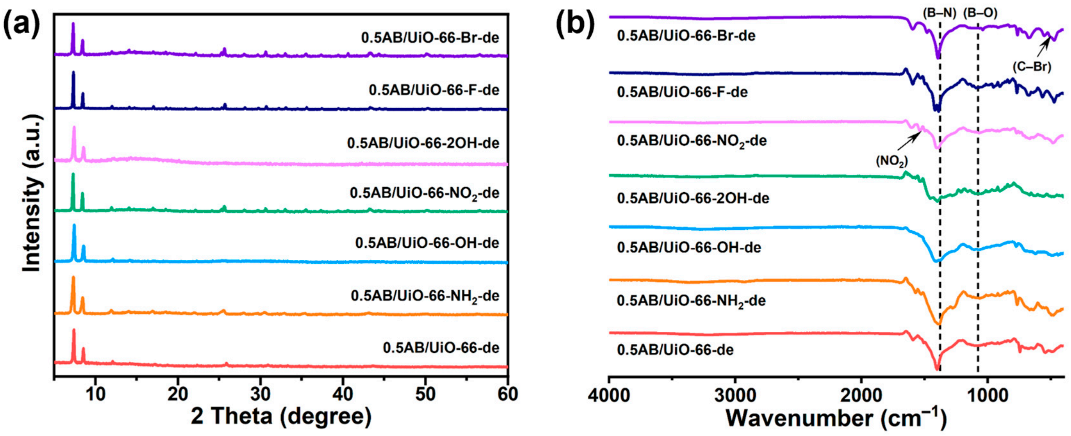

Figure S6. Characterization of 0.5AB/UiO-66-de and 0.5AB/UiO-66-X-de. PXRD patterns (**a**), FT-IR spectra (**b**).

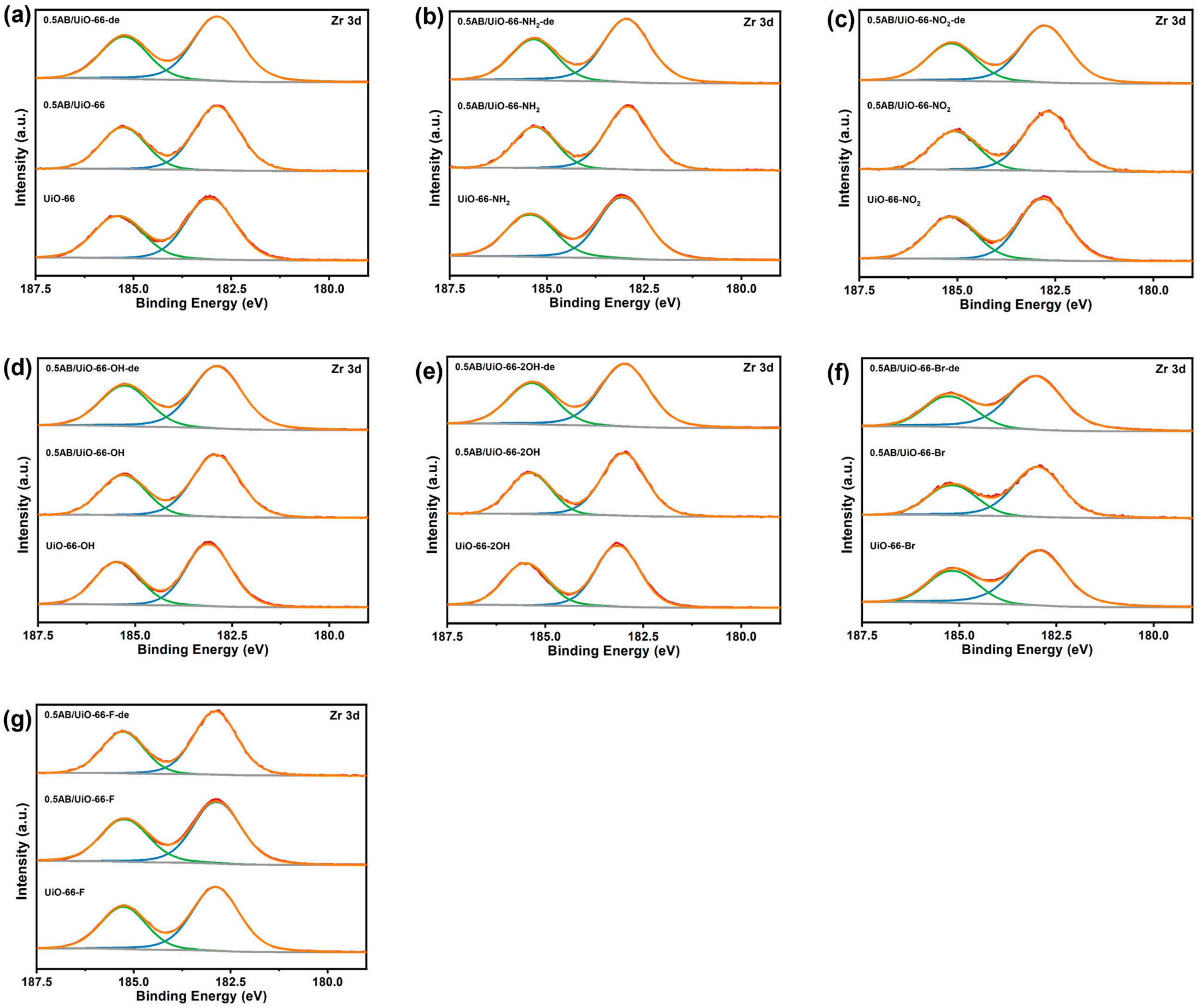

Figure S7. XPS spectra of the Zr 3d orbitals of the materials.

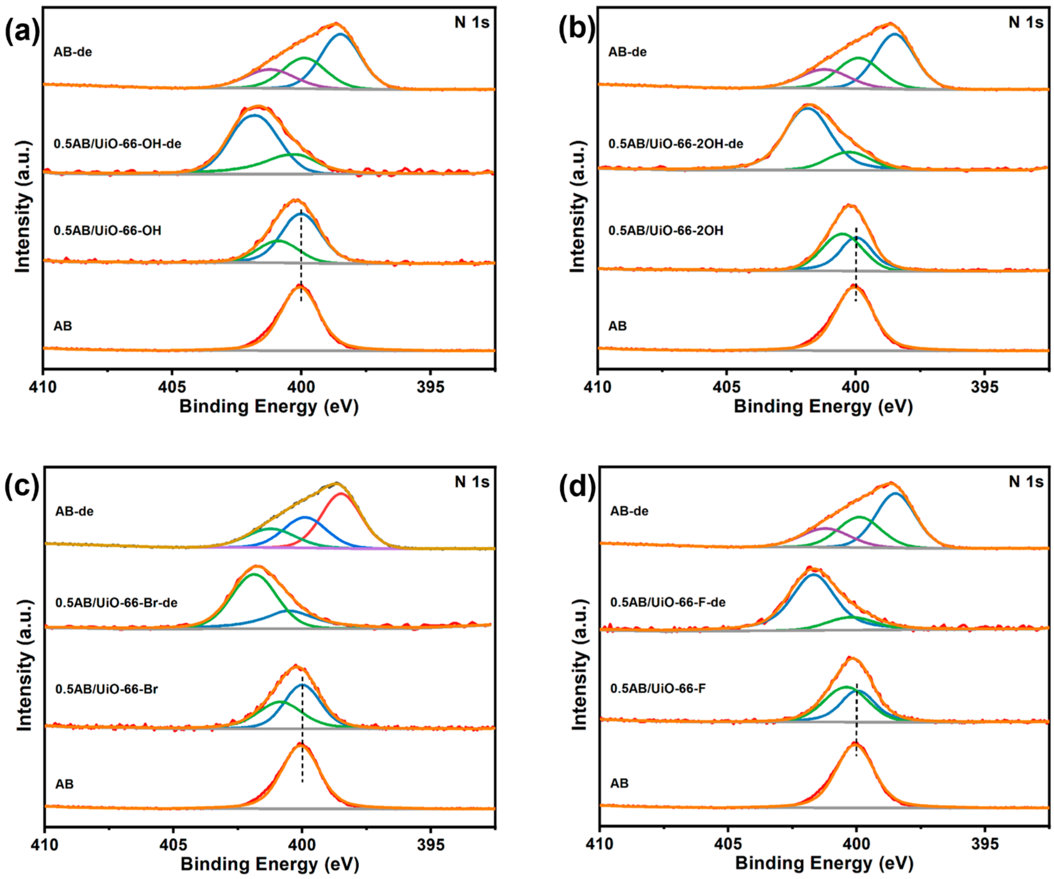

Figure S8. XPS spectra of the N 1s orbitals of the materials.

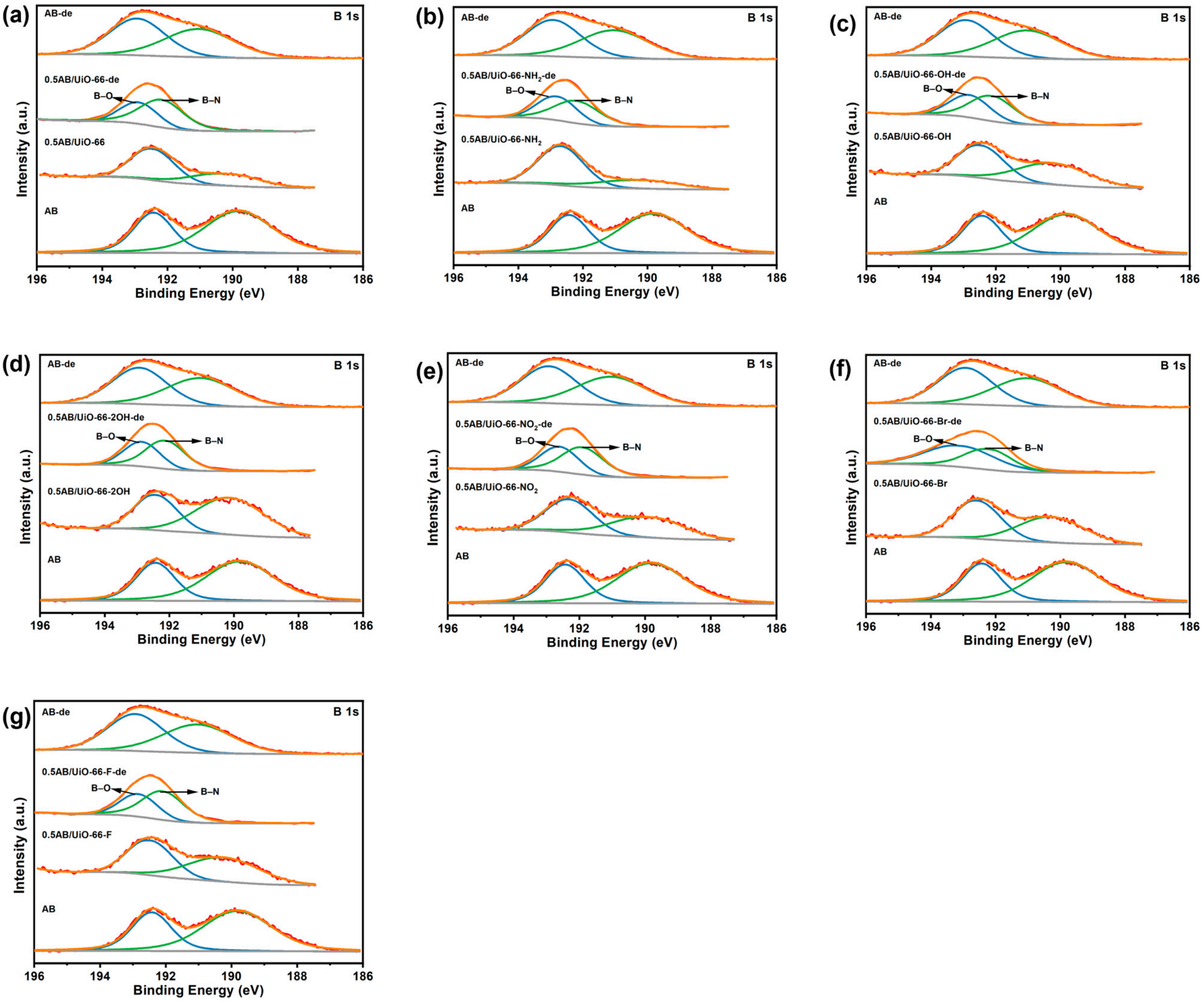

Figure S9. XPS spectra of the B 1s orbitals of the materials.

Table S1. Muliken population analysis of charge densities of AB, H_2BDC, H_2BDC-AB.

	H···NH_3-BH_3		H···BH_3-NH_3	
	Before optimization	After optimization	Before optimization	After optimization
C1	–0.12855	–0.128359	–0.12855	–0.132723
C2	–0.12851	–0.124598	–0.12851	–0.129269
C3	–0.12861	–0.133887	–0.12861	–0.141708
C4	–0.12861	–0.128934	–0.12861	–0.132784
H1	0.128563	0.137445	0.128563	0.117900
C5	–0.12851	–0.116954	–0.12851	–0.142491
H2	0.128552	0.128759	0.128552	0.122122
C6	–0.12855	–0.130445	–0.12855	–0.140708
H3	0.128552	0.137584	0.128552	0.118090
H4	0.128563	0.070671	0.128563	0.200849
H5	–0.08724	–0.086589	**–0.08724**	**–0.076708**
H6	–0.08724	–0.086755	**–0.08724**	**–0.080376**
H7	–0.08724	–0.085960	**–0.08724**	**–0.082939**
H8	**0.36417**	**0.370577**	0.36417	0.366525
H9	**0.36417**	**0.372174**	0.36417	0.366447
H10	**0.36417**	**0.372914**	0.36417	0.366619
B	–0.05357	–0.059971	–0.05357	–0.057283
N	–0.77722	–0.775714	–0.77722	–0.781075
H11	0.128558	0.137990	0.128558	0.118048
H12	0.128558	0.130052	0.128558	0.121465

Table S2. Muliken population analysis of charge densities of AB, NH_2-BDC, NH_2-BDC-AB.

	$NH_2 \cdots NH_3$-BH_3		$NH_2 \cdots BH_3$-NH_3	
	Before optimization	After optimization	Before optimization	After optimization
N1	–0.77722	–0.800419	–0.77722	–0.786782
H1	**0.36417**	**0.406178**	0.36417	0.362043
H2	0.36417	0.347939	0.36417	0.364944
H3	0.36417	0.344641	0.36417	0.362065
B	–0.05357	–0.046758	–0.05357	–0.036648
H4	**–0.08724**	**–0.111543**	**–0.08724**	**–0.078363**
H5	–0.08724	–0.085432	**–0.08724**	**–0.114174**
H6	**–0.08724**	**–0.107199**	**–0.08724**	**–0.078023**
C1	–0.17206	–0.177728	–0.17206	–0.174599
C2	–0.13453	–0.136600	–0.13453	–0.136232
C3	–0.13893	–0.131371	–0.13893	–0.143844
C4	–0.13487	–0.136393	–0.13487	–0.136033
C5	–0.17288	–0.171017	–0.17288	–0.174478
C6	0.306924	0.284639	0.306924	0.340633
H7	0.120093	0.128071	0.120093	0.110927
H8	0.114187	0.119547	0.114187	0.108617
H9	0.124195	0.134455	0.124195	0.115499
N2	–0.78626	–0.852362	–0.78626	–0.795054
H10	0.317904	0.330259	0.317904	0.332689
H11	0.317919	0.375187	0.317919	0.332711
H12	0.114022	0.154814	0.114022	0.115459
H13	0.124282	0.131091	0.124282	0.108645

Table S3. Muliken population analysis of charge densities of AB, OH-BDC, OH-BDC-AB.

	$OH\cdots NH_3\text{-}BH_3$		$OH\cdots BH_3\text{-}NH_3$	
	Before optimization	After optimization	Before optimization	After optimization
N	–0.77722	–0.827799	–0.77722	–0.782075
H1	**0.36417**	**0.417779**	0.36417	0.368449
H2	0.36417	0.348194	0.36417	0.371329
H3	0.36417	0.348194	0.36417	0.369288
B	–0.05357	–0.018207	–0.05357	–0.103870
H4	**–0.08724**	**–0.105351**	**–0.08724**	**–0.046095**
H5	**–0.08724**	**–0.105385**	**–0.08724**	**–0.103055**
H6	**–0.08724**	**–0.097988**	**–0.08724**	**–0.041456**
C1	–0.13315	–0.135177	–0.13315	–0.135340
C2	–0.19458	–0.191840	–0.19458	–0.193276
C3	0.349957	0.342894	0.349957	0.355439
C4	–0.15837	–0.169881	–0.15837	–0.164789
C5	–0.13425	–0.142875	–0.13425	–0.134915
C6	–0.1353	–0.128430	–0.1353	–0.139228
H7	0.130752	0.144284	0.130752	0.119771
H8	0.116518	0.118889	0.116518	0.121783
H9	0.125791	0.131861	0.125791	0.114297
O	–0.6425	–0.686365	–0.6425	–0.644118
H10	0.406374	0.415724	0.406374	0.421511
H11	0.138747	0.207275	0.138747	0.129011
H12	0.130006	0.134203	0.130006	0.117341

Table S4. Muliken population analysis of charge densities of AB, DHTA, DHTA-AB.

	$2OH\cdots NH_3\text{-}BH_3$		$2OH\cdots BH_3\text{-}NH_3$	
	Before optimization	After optimization	Before optimization	After optimization
N	−0.77722	−0.791646	−0.77722	−0.788559
H1	0.36417	0.352167	0.36417	0.365635
H2	0.36417	0.347495	0.36417	0.363302
H3	**0.36417**	**0.396017**	0.36417	0.362164
B	−0.05357	−0.051412	−0.05357	−0.065821
H4	−0.08724	−0.083801	**−0.08724**	**−0.046794**
H5	**−0.08724**	**−0.100758**	**−0.08724**	**−0.059725**
H6	−0.08724	−0.092933	**−0.08724**	**−0.120204**
C1	−0.16402	−0.166083	−0.16402	−0.167096
C2	−0.19811	−0.192696	−0.19811	−0.194247
C3	0.346363	0.333953	0.346363	0.359914
C4	−0.16402	−0.167558	−0.16402	−0.170398
C5	−0.19811	−0.207946	−0.19811	−0.200187
C6	0.346363	0.353923	0.346363	0.342068
H7	0.120013	0.122935	0.120013	0.121057
O1	−0.65058	−0.692697	−0.65058	−0.631625
H8	0.405229	0.412665	0.405229	0.410171
H9	0.120013	0.133039	0.120013	0.110726
O2	−0.65058	−0.646470	−0.65058	−0.656289
H10	0.405229	0.409186	0.405229	0.401175
H11	0.141098	0.186905	0.141098	0.133829
H12	0.141098	0.145714	0.141098	0.130905

Table S5. Muliken population analysis of charge densities of AB, NO_2-BDC, NO_2-BDC-AB.

	NO_2···NH_3-BH_3		NO_2···BH_3-NH_3	
	Before optimization	After optimization	Before optimization	After optimization
N1	−0.77722	−0.793786	−0.77722	−0.796946
H1	**0.36417**	**0.376303**	0.36417	0.354834
H2	**0.36417**	**0.345271**	0.36417	0.358762
H3	**0.36417**	**0.375169**	**0.36417**	**0.378497**
B	−0.05357	−0.045850	−0.05357	−0.041656
H4	−0.08724	−0.093503	**−0.08724**	**−0.096218**
H5	−0.08724	−0.102018	**−0.08724**	**−0.099569**
H6	−0.08724	−0.102357	**−0.08724**	**−0.103422**
C1	−0.14286	−0.138733	−0.14286	−0.140729
C2	−0.11287	−0.105200	−0.11287	−0.106320
C3	−0.14293	−0.138834	−0.14293	−0.141185
C4	−0.11767	−0.144505	−0.11767	−0.132677
C5	0.248467	0.270959	0.248467	0.298154
C6	−0.11762	−0.144426	−0.11762	−0.139654
H7	0.145031	0.159267	0.145031	0.149270
H8	0.162942	0.190704	0.162942	0.183387
H9	0.149585	0.161175	0.149585	0.152166
N2	0.349627	0.372625	0.349627	0.382841
O1	−0.36751	−0.396965	−0.36751	−0.396599
O2	−0.36673	−0.397286	−0.36673	−0.403037
H10	0.162994	0.190869	0.162994	0.189074
H11	0.149534	0.161121	0.149534	0.151027

Table S6. Muliken population analysis of charge densities of AB, Br-BDC, Br-BDC-AB.

	Br···NH_3-BH_3		Br···BH_3-NH_3	
	Before optimization	After optimization	Before optimization	After optimization
N	–0.77722	–0.847967	–0.77722	–0.809696
H1	**0.36417**	**0.411081**	0.36417	0.346467
H2	**0.36417**	**0.410971**	0.36417	0.344134
H3	**0.36417**	**0.411626**	0.36417	0.344189
B	–0.05357	–0.088374	–0.05357	–0.053384
H4	–0.08724	–0.077638	**–0.08724**	**–0.067154**
H5	–0.08724	–0.077315	**–0.08724**	**–0.067382**
H6	–0.08724	–0.077608	**–0.08724**	**–0.063741**
C1	–0.12934	–0.128574	–0.12934	–0.130140
C2	–0.1239	–0.124305	–0.1239	–0.128198
C3	–0.12934	–0.126734	–0.12934	–0.128564
C4	–0.14603	–0.154302	–0.14603	–0.155297
C5	0.080881	0.037862	0.080881	0.051121
C6	–0.14603	–0.156007	–0.14603	–0.154533
H7	0.137309	0.138093	0.137309	0.127862
H8	0.154352	0.144787	0.154352	0.146261
Br	**–0.13413**	**–0.121071**	**–0.13413**	**–0.006825**
H9	0.140944	0.140285	0.140944	0.130963
H10	0.154352	0.144544	0.154352	0.143062
H11	0.140944	0.140644	0.140944	0.130856

Table S7. Muliken population analysis of charge densities of AB, F-BDC, F-BDC-AB.

	F···NH_3-BH_3		F···BH_3-NH_3	
	Before optimization	After optimization	Before optimization	After optimization
N	–0.77722	–0.749917	–0.77722	–0.796584
H1	**0.36417**	**0.359592**	0.36417	0.354587
H2	**0.36417**	**0.357948**	0.36417	0.353672
H3	**0.36417**	**0.358851**	0.36417	0.353488
B	–0.05357	–0.059067	–0.05357	0.005889
H4	–0.08724	–0.100471	**–0.08724**	**–0.098257**
H5	–0.08724	–0.099990	**–0.08724**	**–0.098397**
H6	–0.08724	–0.099802	**–0.08724**	**–0.113684**
C1	–0.12994	–0.131208	–0.12994	–0.132484
C2	–0.13009	–0.124524	–0.13009	–0.130231
C3	–0.12994	–0.130808	–0.12994	–0.131946
C4	–0.19511	–0.188482	–0.19511	–0.189465
C5	0.378182	0.362190	0.378182	0.375028
C6	–0.19511	–0.187573	–0.19511	–0.194087
H7	0.133208	0.144711	0.133208	0.126134
H8	0.145967	0.152417	0.145967	0.160187
H9	0.138306	0.149936	0.138306	0.131410
F	**–0.29976**	**–0.316794**	**–0.29976**	**–0.249931**
H10	0.145967	0.153052	0.145967	0.143402
H11	0.138306	0.149938	0.138306	0.131269

Table S8. The xyz coordinates used for computational analysis.

Atom	x	y	z
	BDC-AB (H···NH_3-BH_3)		
C1	2.198855943	1.271220529	0.041610539
C2	2.993374846	0.126368411	0.129995006
C3	0.815633028	1.150525438	-0.107129687
C4	2.406684227	-1.139658513	0.069320829
H1	4.069758974	0.22072379	0.245721125
C5	0.229347192	-0.115977931	-0.166919799
H2	0.19922764	2.043675566	-0.176348283
C6	1.023813781	-1.261568891	-0.079125004
H3	3.025904917	-2.029959133	0.13784029
H4	-0.850365862	-0.20825662	-0.277902997
H5	-5.156489152	-0.377572548	-0.868519939
H6	-4.86642549	1.26437452	0.290551208
H7	-4.93976162	-0.578558837	1.140658155
H8	-2.777267779	-1.058367537	-0.16777584
H9	-2.719637763	0.440233419	-0.860190176
H10	-2.537893488	0.275665325	0.774944182
B	-4.688149501	0.074553182	0.152632403
N	-3.033859144	-0.079767798	-0.041579748
H11	2.655937252	2.255917485	0.088404088
H12	0.568519799	-2.24772099	-0.125996893
	BDC-AB (H···BH_3-NH_3)		
C1	-2.377231671	-1.215273936	-0.002009765
C2	-3.087168677	-0.01233797	-0.005581834
C3	-0.980426809	-1.201721289	0.004919975
C4	-2.397652844	1.202437033	-0.002089659
H1	-4.174519448	-0.021519421	-0.010939107
C5	-0.287330948	0.011302385	0.008312761
H2	-0.426816275	-2.137471609	0.007799246
C6	-1.000831619	1.212490596	0.004844955
H3	-2.949249925	2.13970369	-0.004800392
H4	0.801534995	0.019703983	0.013636355
H5	2.708733965	0.302721499	-1.145156178
H6	2.705175613	0.85161298	0.810092188
H7	2.689172007	-1.115949472	0.307706831
H8	5.034660537	-0.689809172	-0.662836426
H9	5.048028512	0.906776633	-0.254652398
H10	5.030950322	-0.24483798	0.924135706
B	3.001407079	0.01043048	-0.008218233
N	4.669380573	-0.005783407	0.000383628
H11	-2.912905857	-2.161736646	-0.00465368
H12	-0.463608474	2.157756063	0.00769503
	NH_2-BDC-AB (NH_2···NH_3-BH_3)		
N1	2.979854738	-0.125897118	0.943476446
H1	2.121136744	-0.626018432	0.636009727
H2	2.732667943	0.53879866	1.677507628
H3	3.63445172	-0.791666183	1.355343936
B	3.655616434	0.634332325	-0.344968047

H4	3.866169775	-0.24401354	-1.161120905
H5	2.81765065	1.433741948	-0.727744365
H6	4.666505109	1.174370159	0.044153542
C1	-0.571577169	0.975225451	-0.333226205
C2	-1.839012572	1.465160805	-0.021803391
C3	-2.884202336	0.592751429	0.289488942
C4	-2.647640261	-0.782942807	0.284906826
C5	-1.383559281	-1.283450164	-0.025252878
C6	-0.336381529	-0.406976901	-0.338434593
H7	-3.870294272	0.979581263	0.529237268
H8	-1.206402798	-2.357172758	-0.026142435
H9	-2.009073116	2.538622207	-0.026788448
N2	0.973501757	-0.903197903	-0.584106615
H10	0.964553449	-1.847804412	-0.961917506
H11	1.515765893	-0.305586899	-1.209103634
H12	0.240861028	1.656097735	-0.575232556
H13	-3.451330928	-1.475553105	0.520976945
	NH_2-BDC-AB (NH_2···BH_3-NH_3)		
N1	-5.062917258	0.000280199	0.152795516
H1	-5.454450308	0.822242219	-0.307755298
H2	-5.386031552	0.004738583	1.120465371
H3	-5.454567997	-0.825807027	-0.300214622
B	-3.372737311	-0.000284837	0.08798399
H4	-3.163486667	-0.005785434	-1.10652989
H5	-3.07969689	1.022442376	0.656903179
H6	-3.080107965	-1.017760687	0.666384501
C1	1.339664483	-1.208112167	-0.068036327
C2	2.722424701	-1.201719124	0.095428645
C3	3.429524687	0.000194767	0.178369693
C4	2.722190768	1.201972632	0.095323742
C5	1.33939709	1.208064272	-0.068102913
C6	0.620544749	-0.000119928	-0.153377579
H7	4.508390678	0.000330462	0.303952006
H8	0.80076446	2.151727666	-0.127938088
H9	3.252695363	-2.149446652	0.156121198
N2	-0.765462807	-0.000293552	-0.258476752
H10	-1.188630766	0.835082308	-0.633769661
H11	-1.188510405	-0.835883043	-0.633535051
H12	3.252264882	2.149834227	0.155992125
H13	0.801235406	-2.151880047	-0.127858626
	OH-BDC-AB (OH···NH_3-BH_3)		
N	3.335538649	-0.431455619	-0.000295555
H1	3.806372391	-0.811891327	0.820663609
H2	3.806416265	-0.810913878	-0.821682921
H3	2.367231571	-0.763889161	-0.000514352
B	3.424965672	1.219846096	0.000752046
H4	2.85672531	1.569360033	1.015541577
H5	4.607663843	1.486763767	0.001032797
H6	2.856940045	1.570663476	-1.013716212
C1	-2.838356922	-0.473061482	0.000365159
C2	-1.673653868	-1.243212055	0.000278157

C3	-0.433239698	-0.60509678	-0.000123328
C4	-0.334895938	0.786554294	-0.000431342
C5	-1.508414107	1.5395846	-0.000339858
C6	-2.760980937	0.919526598	0.000054355
H7	-1.73271995	-2.330629846	0.000531834
H8	-1.437820687	2.623681301	-0.000577518
H9	-3.667861717	1.516948186	0.000123235
O	0.748646364	-1.335568053	-0.000207553
H10	0.534147102	-2.281598499	-0.000211112
H11	0.642313726	1.260602455	-0.000721059
H12	-3.804928912	-0.969364289	0.000680335
	OH-BDC-AB (OH···BH_3-NH_3)		
N	-4.56561363	0.46030869	0.333383024
H1	-4.562445943	1.480735454	0.313294606
H2	-4.945769025	0.170563299	1.235119512
H3	-5.207356071	0.135773595	-0.390845135
B	-3.038129779	-0.16504244	0.119843204
H4	-2.72635679	0.260669332	-0.980848552
H5	-2.386905333	0.298260351	1.022239109
H6	-3.184915531	-1.359290763	0.153985075
C1	1.931968398	1.507664046	-0.137303334
C2	0.711287117	0.892575855	-0.417450282
C3	0.578739001	-0.495947442	-0.273329975
C4	1.678117002	-1.254091543	0.153810572
C5	2.89063059	-0.627401505	0.429850908
C6	3.028968334	0.756558215	0.2871356
H7	-0.141093825	1.482611552	-0.744813036
H8	3.736115258	-1.226531049	0.759584267
H9	3.977260823	1.240514468	0.503130809
O	-0.579080114	-1.159628525	-0.532602111
H10	-1.274401466	-0.536667298	-0.80692475
H11	1.558009081	-2.327952834	0.261030672
H12	2.022181418	2.585247676	-0.253313804
	Br-BDC-AB (Br···NH_3-BH_3)		
N	-3.107490597	0.000186815	0.00146916
H1	-2.91268328	-0.822410873	0.54949448
H2	-2.912491384	0.88529889	0.44134554
H3	-2.910772574	-0.062061248	-0.984703524
B	-4.853627857	0.000316679	-0.000850219
H4	-5.136674586	-1.051742915	-0.524510954
H5	-5.139005337	0.073349531	1.171488646
H6	-5.136350915	0.979498272	-0.650830198
C1	3.12193359	1.208258776	0.000079609
C2	3.821864468	0.000358749	0.000257211
C3	3.122352598	-1.207872789	0.000089181
C4	1.72575231	-1.213148279	-0.000157392
C5	1.039517561	-0.000045461	-0.000213476
C6	1.725483509	1.213119364	-0.000146842
H7	4.908175231	0.000466435	0.000504033
H8	1.179377922	2.15152135	-0.000403534
Br	-0.907492356	-0.00027236	-0.000221112

H9	3.661277906	-2.151530431	0.000150506
H10	1.17991881	-2.151765454	-0.000410062
H11	3.66060983	2.151995805	0.000131194
	Br-BDC-AB (Br···BH_3-NH_3)		
N	4.769681977	0.461488073	-0.012166823
H1	5.026235993	1.446683396	-0.072727605
H2	5.190737228	-0.01093669	-0.812963874
H3	5.208352025	0.084813572	0.828549239
B	2.943822546	0.278039296	0.026831722
H4	2.801125884	0.859306852	1.06555024
H5	2.753492698	0.881455396	-0.995648322
H6	2.994923589	-0.928685529	0.013438717
C1	-2.911677361	1.475018906	-0.000389983
C2	-3.805303723	0.401810998	0.002440664
C3	-3.316051823	-0.905786331	0.000143132
C4	-1.939621041	-1.145776167	-0.00264302
C5	-1.058131582	-0.065192715	-0.003314609
C6	-1.533956959	1.245910219	-0.003588219
H7	-4.875975922	0.582826688	0.005419976
H8	-0.837378329	2.077567692	-0.006935712
Br	0.853311379	-0.406800579	-0.000704604
H9	-4.004616482	-1.746237296	0.000698447
H10	-1.556933882	-2.160826509	-0.00528958
H11	-3.284292642	2.495530277	-0.000309032
	F-BDC-AB (F···NH_3-BH_3)		
N	3.111819843	0.004857391	0.00244342
H1	2.739396633	-0.285416313	-0.896041532
H2	2.755759995	0.934733168	0.198943118
H3	2.741753255	-0.622887925	0.709023254
B	4.760574779	-0.011899242	-0.003067291
H4	5.069002288	-1.160755455	-0.245835134
H5	5.086998151	0.766682741	-0.875390961
H6	5.089522305	0.34802269	1.108647604
C1	-2.527263243	1.202206332	-0.000456881
C2	-3.216391338	-0.012598701	-0.000648012
C3	-2.509379613	-1.217111171	-0.000421844
C4	-1.112781473	-1.216842237	0.000057324
C5	-0.460831198	0.007878298	0.000413212
C6	-1.130849072	1.222614566	0.000062953
H7	-4.30211174	-0.020675896	-0.000998876
H8	-0.571554504	2.152499259	-0.000077195
H9	-3.042269035	-2.163456355	-0.000602705
F	0.911852475	0.017522235	0.000810006
H10	-0.539657864	-2.138350499	-0.000020303
H11	-3.0741489	2.140516411	-0.000745319
	F-BDC-AB (F···BH_3-NH_3)		
N	-4.607119764	0.276686122	-0.029882574
H1	-5.165481006	-0.570003771	0.07593341
H2	-4.873044376	0.716671601	-0.91055834
H3	-4.860265131	0.91279539	0.725875888

B	-2.905270612	-0.095992762	0.000761641
H4	-2.814255078	-0.591202733	1.097151149
H5	-2.831704111	-0.836196951	-0.949011984
H6	-2.440707747	1.012625096	-0.136195363
C1	2.13687085	1.440970409	0.001166773
C2	3.154646137	0.484043621	-0.024048574
C3	2.829338419	-0.874469442	-0.026545049
C4	1.494337813	-1.281533854	-0.004032605
C5	0.501356229	-0.307412591	0.021356915
C6	0.796454438	1.052029137	0.024193987
H7	4.195302678	0.795287242	-0.041660683
H8	-0.014685325	1.771408442	0.043266921
H9	3.616206361	-1.623883038	-0.046072598
F	-0.790114794	-0.699520713	0.044044846
H10	1.212588144	-2.329535021	-0.005260084
H11	2.385246897	2.499117416	0.002949206
NO_2-BDC-AB (NO_2···NH_3-BH_3)			
N1	3.240460737	0.002885734	-0.489680534
H1	2.99817478	-0.790057013	0.103605001
H2	2.631973233	-0.028655223	-1.307150568
H3	2.998755912	0.838540667	0.041622461
B	4.829476461	-0.01360076	-0.954487926
H4	5.465505932	0.027710193	0.076941314
H5	4.968614755	-1.05230597	-1.567002152
H6	4.966209994	0.974398575	-1.6464241
C1	-2.820246249	1.208598735	-0.426869406
C2	-3.470530669	-0.007110117	-0.659137255
C3	-2.81535519	-1.218195931	-0.416880796
C4	-1.506382088	-1.223301147	0.05783094
C5	-0.876717974	0.00196621	0.280633955
C6	-1.511259663	1.222868016	0.047668834
H7	-4.491543018	-0.010626028	-1.029689686
H8	-0.983233866	2.149064825	0.23735551
H9	-3.323757814	-2.160168482	-0.597607593
N2	0.504741123	0.006969235	0.773072667
O1	1.046155449	-1.082382336	0.977894664
O2	1.043297929	1.099995699	0.964297872
H10	-0.974701063	-2.145774998	0.255214092
H11	-3.332470193	2.147030969	-0.615187518
NO_2-BDC-AB (NO_2···BH_3-NH_3)			
N1	3.545448039	0.244916828	-0.436718403
H1	4.353529945	0.847689083	-0.284331977
H2	3.88296448	-0.651367594	-0.786752889
H3	3.09240051	0.080108997	0.468206541
B	2.479976869	0.944265412	-1.490031818
H4	2.09396345	1.951351647	-0.936815041
H5	3.126562902	1.169591779	-2.492090969
H6	1.611077954	0.123946255	-1.677487916
C1	-2.661018031	-0.88536065	-0.758282835
C2	-3.216042139	0.364470205	-0.468278423
C3	-2.469290267	1.328651934	0.214587992

C4	-1.165499222	1.049665762	0.616101947
C5	-0.63434666	-0.204435701	0.317985885
C6	-1.357823751	-1.179966854	-0.367005606
H7	-4.232247849	0.589170645	-0.779742422
H8	-0.897205664	-2.135500932	-0.583804566
H9	-2.900178758	2.301409049	0.430854228
N2	0.730037179	-0.510894802	0.741544858
O1	1.217836223	-1.594190728	0.422337685
O2	1.32780724	0.328901587	1.425667672
H10	-0.558054536	1.778685857	1.136774582
H11	-3.242120592	-1.630401042	-1.293132293
	DHTA-AB (2OH···NH_3-BH_3)		
N	3.722543074	0.156687554	-0.019722987
H1	3.656295506	0.432999882	-0.998821354
H2	4.548122141	0.608747192	0.373101042
H3	2.887707992	0.529569322	0.430686486
B	3.863349064	-1.483129108	0.130683305
H4	2.873332823	-1.949569521	-0.398234854
H5	4.883293936	-1.777476163	-0.455688813
H6	3.911521006	-1.692659115	1.324118187
C1	-2.323025172	0.966614891	0.071343566
C2	-1.061722992	1.559454883	0.048693448
C3	0.081618421	0.764781867	-0.036844678
C4	-0.024139344	-0.62313985	-0.0992438
C5	-1.287070165	-1.212444886	-0.07567019
C6	-2.440057605	-0.424433039	0.00926772
H7	-0.975863147	2.643868175	0.097119795
O1	1.359845488	1.320478765	-0.066244177
H8	1.273222141	2.28412811	0.009959387
H9	-1.3700812	-2.296553435	-0.122836648
O2	-3.703268687	-0.953911679	0.035680823
H10	-3.639746763	-1.920429783	-0.008168964
H11	0.873269199	-1.232201983	-0.164978423
H12	-3.221853754	1.570870099	0.137618975
	DHTA-AB (2OH···BH_3-NH_3)		
N	4.857415327	-0.609887199	-0.629144097
H1	5.632797805	-0.231752505	-0.084398377
H2	5.029218578	-0.384721783	-1.609205434
H3	4.876368852	-1.625864394	-0.537126909
B	3.373907428	0.041398522	-0.12905499
H4	2.58383454	-0.510691355	-0.851961559
H5	3.523910096	1.222086797	-0.289740798
H6	3.351178937	-0.319235688	1.037847509
C1	-1.712987015	-1.212464777	0.363162878
C2	-0.417655264	-0.811038378	0.685874956
C3	-0.00082219	0.507118309	0.455747687
C4	-0.903087628	1.415206594	-0.108305635
C5	-2.198527694	1.01372634	-0.430640243
C6	-2.610700165	-0.301662273	-0.197247488
H7	0.275452587	-1.527189813	1.12134108
O1	1.254149159	0.954758754	0.749050179

H8	1.793330398	0.233679528	1.113138091
H9	-2.891628343	1.731248112	-0.867925847
O2	-3.877109922	-0.751945461	-0.496123772
H10	-4.383387793	-0.015406738	-0.870997817
H11	-0.575981368	2.434545007	-0.288144362
H12	-2.04017291	-2.232300612	0.541493839
